# Medfly Gut Microbiota and Enhancement of the Sterile Insect Technique: Similarities and Differences of *Klebsiella oxytoca* and *Enterobacter* sp. AA26 Probiotics during the Larval and Adult Stages of the VIENNA 8^D53+^ Genetic Sexing Strain

**DOI:** 10.3389/fmicb.2017.02064

**Published:** 2017-10-27

**Authors:** Georgios A. Kyritsis, Antonios A. Augustinos, Carlos Cáceres, Kostas Bourtzis

**Affiliations:** Insect Pest Control Laboratory, Joint FAO/IAEA Programme of Nuclear Techniques in Food and Agriculture, Vienna, Austria

**Keywords:** *Ceratitis capitata*, tephritids, insect symbiosis

## Abstract

The Mediterranean fruit fly, *Ceratitis capitata*, is a major agricultural pest worldwide. The development of genetic sexing strains (GSSs) for this species that allows male-only sterile insects releases has boosted the effectiveness of the environmental friendly pest control method known as the sterile insect technique. The last generation of these strains, the VIENNA 7 and VIENNA 8, are currently used in all mass rearing facilities worldwide and are considered as models for such pest control applications. The sterile insect technique depends on the rearing of sufficient numbers of adequate “biological quality” laboratory flies to be released in the field. Currently, there is an increasing amount of studies focusing on the characterization of the symbiotic communities and development of probiotic diets. In our study, two bacterial isolates, an *Enterobacter* sp. (strain AA26) and a *Klebsiella oxytoca* strain, were used as probiotics in larval and adult diet. These strains have been shown to be beneficial, affecting several aspects related to the rearing efficiency and biological quality of the medfly VIENNA 8^D53+^ GSS. Our results demonstrate the effect of *K. oxytoca* on the developmental duration of the immature stages and, to some extent, on flight ability. On the other hand, our study does not support the presence of any beneficial effect of (a) *K. oxytoca* on pupal and adult recovery and adults’ survival under stress conditions when provided as a larval diet supplement and (b) *K. oxytoca* and *Enterobacter* sp. AA26 on mating competitiveness when provided as adult diet supplements. Possible explanations for inconsistencies with previous studies and the need for universalizing protocols are discussed. Our findings, combined with previous studies can support the sterile insect technique, through the improvement of different aspects of mass rearing and biological properties of laboratory reared insect pests.

## Introduction

The Mediterranean fruit fly, *Ceratitis capitata* (Diptera: Tephritidae), is a cosmopolitan species that poses severe threats to a variety of fruits worldwide (White and Elson-Harris, 1992). Due to its economic importance, it has been a target species for Integrated Pest Management (IPM) for many years. The extensively studied biology of *C. capitata* made it a Tephritidae model for the implementation of area-wide control methods, like Sterile Insect Technique (SIT). SIT relies on the mass rearing and release of sterile flies intended to mate with the natural population, causing infertile crosses and subsequent population suppression (Dyck et al., 2005). In most of the cases, in the SIT approach, sterility is delivered through irradiation. The implementation of a large-scale SIT program depends on the feasibility to produce (a) the adequate number of insects in a sustainable cost, and (b) high biological quality insects showing adequate performance and mating competitiveness in the field.

In the case of medfly, a breakthrough in SIT applications has been the development of genetic sexing strains (GSSs). SIT in medfly has been shown to be more effective when only males are released, compared to bisexual releases (Hendrichs et al., 1995). This is because, (i) the mass reared released males may mate with the mass-reared released females and (ii) the released females, although sterile, could oviposit in fruits mesocarp causing considerable damage. The development and utilization of the GSS offers the advantage of male only releases (females are temperature-sensitive and could be killed when exposed to heat treatment during the last stage of the embryonic development) and consequently reduces the rearing cost (Fisher, 1998, 2000; Cáceres, 2002; Franz, 2005). The VIENNA 7 and VIENNA 8 medfly GSS are currently used in all mass rearing facilities worldwide^1^ (Augustinos et al., 2017). VIENNA 8 may also incorporate the D53 inversion (VIENNA 8^D53+^) that leads to restriction in the recombination and improved stability of the genetic sexing system (Franz, 2005; Augustinos et al., 2017).

Symbiotic relationships are recently receiving much attention, since, it has been shown that they can influence host’s fitness and behavior. Therefore, characterization of symbiotic communities and development of probiotic diets are potential tools to improve insects’ quality and make them more effective for SIT applications. Medfly is also a Tephritidae model for the application of symbionts in delivering better laboratory strains, since many recent studies have tried to: (a) characterize its gut symbiotic communities (Behar et al., 2008; Ben Ami et al., 2010; Aharon et al., 2012; Hamden et al., 2013; Augustinos et al., 2015), (b) assess the potential impact of probiotic additives on larval development and possible downstream benefits (Ben Ami et al., 2010; Hamden et al., 2013; Augustinos et al., 2015) and, (c) test whether the provision of bacterial enriched diets in adult stages prior to release could improve the released males sexual performance and consequently the SIT effectiveness (for example, provide more robust or competitive males) (Niyazi et al., 2004; Ben Ami et al., 2010; Gavriel et al., 2011).

Medfly is an ideal tephritid species for such studies, due to the established SIT operational programs, the existence of the GSS that are universally used in all SIT operational programs, and the extended research that managed to optimize mass rearing protocols, combining cost effective and less labor intensive practices (Fisher, 1998, 2000; Cáceres, 2002; Franz, 2005). However, the cost of the larval diet and the sexual competitiveness of released males are parameters that could be optimized even more. For the above reasons, medfly GSS are ideal for controlled and reproducible probiotic experiments where any beneficial action of bacterial enriched diets can be relatively quickly employed by the mass-rearing facilities.

In the previous years, Ben Ami et al. (2010) isolated, among others, a medfly gut symbiont identified as *Klebsiella oxytoca*, during a screening for cultivable bacteria in the gut of medfly. Their findings suggest that feeding sterile VIENNA 8 GSS males (VIENNA 8^D53+^) with *K. oxytoca* shorten their mating latency time and this effect is more pronounced in the case of provisioning live than autoclaved bacteria. The same research group extended their study on the probiotic effects of *K. oxytoca* by recording different biological quality components on VIENNA 8^D53+^ adults (Gavriel et al., 2011). The findings of this study showed that: (a) the addition of bacteria to sterile male diet significantly increased their mating success, both with “wildish” females in laboratory conditions and with “wild” females in semi-natural conditions, (b) bacterially fed sterile males were more effective than sugar fed males in inhibiting female receptivity and, (c) sterile males fed on bacteria enriched diet showed better ability to survive after 48 and 72 h of starvation, compared to the sugar-only fed. Later, Hamden et al. (2013) used a ‘probiotic cocktail’ as a supplement to larval diet of VIENNA 8 males. This mix consisted of three different bacterial species, provided by the Pasteur Institute (*Klebsiella pneumoniae, Citrobacter freundii*, and *Enterobacter cloacae*), which were not medfly or other insects’ isolates. This study pointed out that the bacteria provision improved the male mating competitiveness of the sterile VIENNA 8 GSS, as well as male longevity under food deprivation. On the other hand, no effects were recorded on pupal weight, flight ability, and adult recovery rates. More recently, an *Enterobacter* sp. (named strain AA26) was isolated from the VIENNA 8^D53+^ gut and was used as a medfly probiotic (Augustinos et al., 2015). Incorporating this isolate in VIENNA 8^D53+^ larval diet resulted in faster immature development (more pronounced in the case of live compared to autoclaved bacteria provision) and decreased mortality during immature stages. The same study did not detect any bacterial effect on pupal weight, male mating competitiveness, flight ability, and adults’ ability to survive under stress conditions. Probiotic diets have been also tested in only few other Tephritidae pests restricted to two *Bactrocera* sp., *B. oleae* (Sacchetti et al., 2014) and *B. tryoni* (Meats et al., 2009), *Zeugodacus* sp. (*Z. tau*) (Khan et al., 2014), and *Anastrepha* sp. (*A. obliqua*) (Rull et al., 2015).

In the present study, we aimed to (a) use and evaluate the *K. oxytoca* isolate as a probiotic in the medfly VIENNA 8^D53+^ larval diet and (b) use and compare the *Enterobacter* sp. AA26 and *K. oxytoca* (discussed above) effects on VIENNA 8^D53+^ male mating competitiveness, when provided through the adult diet. Both targets were approached following the standard quality control (QC) procedures established for the evaluation of sterile insects in SIT operational programs.

## Materials and Methods

### Medfly Strains and Rearing Conditions

The experiments were conducted at the Joint FAO/IAEA Insect Pest Control Laboratory (hereafter IPCL), Seibersdorf, Austria, using the VIENNA 8^D53+^ GSS medfly colony reared in the IPCL (Franz, 2005; Augustinos et al., 2016, 2017). Adult flies were kept in two-side fine mesh cages provided with water and adult diet consisting of sugar and yeast hydrolyzate at a 3:1 ratio (Cáceres, 2002). Eggs were collected in water containers placed below a mesh cover.

### Origin and Characterization of Gut Symbionts Used in This Study

The *Enterobacter* sp. AA26 strain used in this study was previously isolated and used as a medfly larval probiotic (Augustinos et al., 2015). The *K. oxytoca* strain has been isolated, described and used as a medfly probiotic in different studies (Ben Ami et al., 2010; Gavriel et al., 2011). Prior to the experiments, both strains were revived from glycerol stocks kept in -80°C, through striking in Petri dishes containing LB agar. A single colony was selected to (a) start the liquid cultures needed for all downstream experiments and, (b) confirm their identity by sequencing the full 16S rRNA gene.

Polymerase chain reactions (PCRs) were performed on individual bacterial colonies for the almost complete amplification of the 16S *rRNA* gene using the universal bacterial primers 27F/1492R (Edwards et al., 1989; Weisburg et al., 1991) as follows: a small number of bacteria was taken from a colony using a sterile tip and was suspended in 50 μl PCR reaction [25 μl of Qiagen 2x Taq mix, 0.3 μl (100 μM stock) of each primer]. PCR conditions were: initial denaturing step of 95°C, for 5 min, followed by 35 cycles of denaturation at 95°C for 45 s, annealing at 55°C for 1 min and extension at 72°C for 2 min. A final extension step of 72°C for 10 min was used. Five μl of each reaction were electrophoresed on 1.5% agarose gels. The amplicons were purified using the High Pure PCR Product Purification Kit (Roche, Germany). Purified DNA was sequenced from both ends using primers 27F and 1492R (Edwards et al., 1989; Weisburg et al., 1991), as well as the internal primers 519F, 596R, 960R, and 1114F (Reed et al., 2002). Sequencing was performed by MWG Eurofins (Germany) and/or VBC (Austria). Electropherograms were visualized, checked for data quality and assembled using the SeqMan software (Lasergene 7.0; Dnastar, Inc.).

### Preparation of Probiotic Enriched Larval Diet

The preparation of larval diet was performed as described in Augustinos et al. (2015). More specifically, 10^6^, 10^7^, and 10^8^ bacteria (grown in LB broth) per gram of larval diet were used. To distinguish between bacteria having an effect either through interaction with the insects or just as nutrient source, both autoclaved and live bacteria of the same concentration were used. To minimize additional manipulation of bacteria prior to experiments, we did not wash them to remove the LB medium. Instead, as a control, larval diet with the same volume of LB medium (20 ml) incorporated, was used.

### Egg Collections

Eggs laid during a period of 6 h were collected from the beginning of the photoperiod at 7:30 am. The eggs were placed on moist filter paper resting on wet sponges infused with 0.3% propionic acid. For the immature survival and developmental experiment (**Supplementary Figure S1**) and to measure pupal weight, 24 h after egg collection, filter papers with 300 eggs each were transferred to a Petri dish (70 mm × 15 mm) with 150 g carrot diet for larval development (Tanaka et al., 1969). For mating competitiveness, adult survival under stress and flight ability assessments, approximately 7000 eggs were placed on 29 cm × 9 cm × 2 cm trays containing 500 g of carrot diet. The bacteria-enriched carrot diet was prepared by hand mixing 1 kg of carrot diet with the respective bacterial suspension before filling the Petri dishes or trays.

### Effects of *K. oxytoca* as a Larval Diet Additive on Medfly’s Immature Survival and Development

Eggs were transferred into carrot diet following the procedure described above. The number of pupae formed was recorded daily by sieving the sawdust, which was used as pupation substrate. Pupae were transferred to Petri dishes where the adult emergence was also recorded daily. The egg to pupae developmental duration and the pupal stage duration were also recorded. Three replicates per treatment were performed, with 300 eggs each. Development of immature stages took place under controlled temperature, humidity, and lighting conditions (22°C, 65 ± 2% RH, 14 h L: 10 h D).

### Effects of *K. oxytoca* as a Larval Diet Additive on Medfly’s Longevity under Food and Water Deprivation

Within 4 h of adult emergence (07:30–11:30 am), 30 males and 30 females were placed in a large Petri dish (70 mm × 15 mm) with a mesh-covered window in the lid and a hole of approximately 15 mm in the center of the lid. All dishes were kept in the dark at 26°C and 65% RH, until the death of the last fly. Dead flies were sorted by sex, counted and removed from the Petri dishes on inspection twice a day (every 12 h; at 19:30 pm and 07:30 am). Three replicates for each treatment [“without bacteria,” “autoclaved bacteria,” or “live bacteria” (10^8^ bacteria per gram of larval diet)] were performed as previously described (Augustinos et al., 2015).

### Effects of *K. oxytoca* as a Larval Diet Additive on Medfly’s Flight Ability

Two days before emergence, 50 male and 50 female pupae (brown and white, respectively) were placed within a ring of paper, which was centered in the bottom of a Petri dish (77 mm × 15 mm). One black plexiglass tube was placed over the Petri dish, following the procedure described in detail in FAO/IAEA/USDA (2014). Flies that emerged were removed from the vicinity of the tubes to minimize fly-back (or fall-back) into the tubes. The flight ability test took place at 26°C and 65% RH, 14 h L: 10 h D and 1500 lux light intensity over the tubes. Three replicates (three tubes with 50 pupae each) were set up per treatment [“without bacteria,” “autoclaved bacteria,” or “live bacteria” (10^8^ bacteria per gram of larval diet)] and sex as previously described (Augustinos et al., 2015).

### Effects of *Enterobacter* sp. AA26 and *K. oxytoca* As Adult Diet Additives on Male Mating Competitiveness

VIENNA 8^D53+^ males were fed onto two different sugar based diets, provisioned either with the *Enterobacter* sp. 26 or with *K. oxytoca.* VIENNA 8^D53+^ males (control and fed on the different probiotic diets) were competing for mating with females either from the VIENNA 8^D53+^ strain or from a recently colonized population deriving from Australia (females of the fourth generation in the lab were used). VIENNA 8^D53+^ males fed on Sugar/Bacteria diets (*K. oxytoca* or *Enterobacter* sp. AA26) were competing against VIENNA 8^D53+^ Sugar/LB fed males (using the same volume of LB). Males used in the experiment were sterilized at pupal stage (24–48 h before emergence) by exposing the pupae to 100 Gy using a gamma beam source (G-220). Females were fed on an adult diet consisting of a mixture of sugar and protein (yeast hydrolysate) at a 3:1 ratio, whereas males were assigned to one of the following diets: Sugar/LB, Sugar/*Enterobacter* sp. 26, Sugar/*K. oxytoca.* Adult food was provided *ad libitum*. A round cotton pad (2.5 cm radial) was soaked with 5 ml of: (i) LB medium, (ii) *Enterobacter* sp. AA26 culture (10^8^ bacteria/ml), (iii) *K. oxytoca* culture (10^8^ bacteria/ml) and put into the males’ cage. The cotton pads were replaced daily. Mating tests were conducted in 45 cm × 45 cm× 45 cm cages (91 lt), under controlled temperature and humidity conditions (26 ± 1°C, 45–55% RH). One day before testing, the males were marked on the thorax with a dot of a non-toxic dye (either yellow or red). The color was rotated between the replicates in order to eliminate bias. On testing days, 25 females (aged 6–8 days) and 25 males of each of the two groups [Sugar/LB and Sugar/Bacteria (*Enterobacter* sp. AA26 or *K. oxytoca*)] (50 males per cage in total, aged 5–6 days) were released in the experimental cages. The cages were visually inspected every 15 min, during 08:00–10:00 am. Mating couples were gently removed and placed into transparent plastic vials and maintained there until the end of copulation. We recorded the total number of females that achieved mating and the type of their male mating partner (bacteria fed or not). Seven replicates (experimental cages) were performed with VIENNA 8^D53+^ females and four replicates with Australian females, for each of the two treatments (Sugar/LB vs. Sugar/*Enterobacter* sp. AA26 and Sugar/LB vs. Sugar/*K. oxytoca*).

### Statistical Analysis

Data analyses were performed using SPSS 20.0 (SPSS, Inc., Chicago, IL, United States). The effects of *K. oxytoca* provision and *K. oxytoca* concentration on pupal recovery, adult emergence and sex ratio of the emerged flies were estimated using binary logistic regression analysis. As the concentration of bacteria, either autoclaved or live, was not a significant predictor (*P* > 0.05) neither for pupal and adult recovery rates nor for adult sex ratio, it was removed from the final model. Binary logistic regression analysis was also used to infer the effects of probiotics (*K. oxytoca*) provision and gender on adult flight ability. Kaplan–Meier estimators of immature developmental times (pupation day, pupal stage duration, and total immature stages duration) were calculated to determine the effects of *K. oxytoca* addition in the larval diet. Pair-wise comparisons between the different bacteria treatments were conducted using log-rank (Mantel–Cox) test. The effects of probiotic provision and gender on adult survival under stress conditions were determined by Cox regression analysis. Chi-square test (x^2^) was used to determine the effects of bacteria provision on male mating competitiveness.

## Results

### Effect of *K. oxytoca* as a Larval Diet Additive on Developmental and Rearing Efficiency Parameters

#### Egg to Pupae Recovery

**FIGURE 1:**
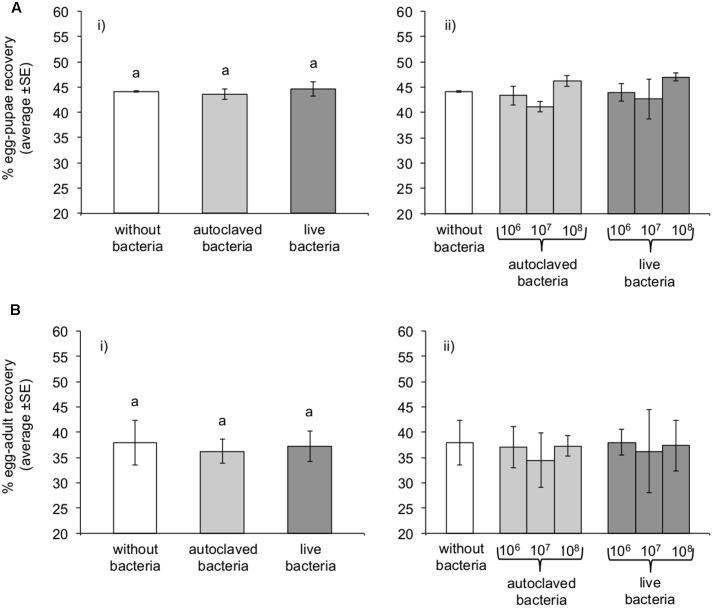
Effect of *Klebsiella oxytoca* incorporation in larval diet on pupae **(A)** and adult **(B)** recovery rates. (i) Irrespective of the *K. oxytoca* concentration, or (ii) considering the three different *K. oxytoca* concentrations as different treatments. Columns marked on top with the same letter are not significantly different (*P* > 0.05).

Logistic regression analysis revealed that the bacterial concentration (10^6^, 10^7^, or 10^8^ bacteria per gram of larval diet) was not a significant predictor of egg to pupae recovery rates neither for “autoclaved” (Wald’s test *t* = 3.57, df = 1, *P* = 0.059) nor for “live” bacteria (Wald’s test *t* = 3.07, df = 1, *P* = 0.079). Therefore, considering that the three bacterial concentrations exert the same effect on recovery rates within each treatment (“autoclaved” and “live bacteria”), we combined the data for the three different concentrations, forming three discrete treatments (“without bacteria,” “autoclaved bacteria,” and “live bacteria”). The provision of *K. oxytoca* was not a significant predictor of the pupal recovery rate (Wald’s test *t* = 0.55, df = 2, *P* = 0.760). The addition of “live bacteria” in the diet did not modify the pupal recovery rate over both control and “autoclaved bacteria” (Wald’s test *t* = 0.05, 0.55, df = 1, *P* = 0.816, 0.459, respectively). The addition of “autoclaved bacteria” had no effect comparing to “without bacteria” treatment (Wald’s test *t* = 0.08, df = 1, *P* = 0.771) (**Figure 1A**).

#### Egg to Adult Recovery

The bacteria concentration did not have a statistically significant effect on adult recovery rates neither for “live” (Wald’s test *t* = 0.002, df = 1, *P* = 0.962) nor for “autoclaved” (Wald’s test *t* = 0.448, df = 1, *P* = 0.503) bacteria diets. Logistic regression analysis revealed that the provision of *K. oxytoca* was not a significant predictor of the adult recovery rates (Wald’s test *t* = 1.00, df = 2, *P* = 0.606). The provision of “live bacteria” did not modify adult emergence rates compared to both “without bacteria” (Wald’s test *t* = 0.14, df = 1, *P* = 0.705) and “autoclaved bacteria” diets (Wald’s test *t* = 0.54, df = 1, *P* = 0.463). The “autoclaved bacteria” diet had also no apparent differential effect on the adult recovery rates compared to the control treatment (Wald’s test *t* = 0.81, df = 1, *P* = 0.369) (**Figure 1B**).

#### Adult Sex Ratio

Logistic regression did not detect any differential effect among the three bacteria concentrations on the sex ratio (Wald’s test *t* = 0.92, df = 2, *P* = 0.631). Specifically, bacteria concentration was not a significant predictor of the adult sex ratio neither for “autoclaved” nor for “live bacteria” (Wald’s test *t* = 0.37, df = 1, *P* = 0.54, and *t* = 0.56, df = 1, *P* = 0.455, respectively). Similarly, bacterial provision also did not alter the sex ratio (Wald’s test *t* = 1.02, df = 2, *P* = 0.600). Pairwise comparisons between “live bacteria” and control, “live bacteria” and “autoclaved bacteria,” and “autoclaved bacteria” and control did not reveal significant differences (Wald’s test *t* = 1.02, df = 1, *P* = 0.312; Wald’s test *t* = 0.13, df = 1, *P* = 0.720; Wald’s test *t* = 1.57, df = 1, *P* = 0.452, respectively) (**Supplementary Figure S1**).

#### Larval Developmental Duration

Data analysis revealed that bacteria provision, either “autoclaved” or “live,” reduced the pre-pupa developmental duration for both sexes compared to the control treatment (“autoclaved”: log rank test, x^2^ = 13.98, *P* < 0.0001 for males and x^2^ = 15.38, *P* < 0.0001 for females; “live”: x^2^ = 15.68, *P* < 0.0001 for males and x^2^ = 17.75, *P* < 0.0001 for females). The two bacteria enhanced diets, consisted of “autoclaved” or “live bacteria,” exerted the same effect on the larval developmental duration (x^2^ = 0.17, *P* = 0.680 for males, x^2^ = 0.795, *P* = 0.07 for females) (Supplementary Table S1 and **Figure S2A**).

#### Pupal Developmental Duration

Bacterial addition in larval diet, either “autoclaved” or “live,” did not affect the females’ pupal stage duration (*P* > 0.05) (Supplementary Table S2 and **Figure S2B**). In contrast to that obtained with females, “autoclaved bacteria” statistically significant reduced the pupal stage duration of the males compared to control treatment (log rank test x^2^ = 11.22, *P* = 0.001) although we did not detect statistically significant differences between “live bacteria” and control (x^2^ = 2.99, *P* = 0.083). “Autoclaved bacteria” significantly reduced the males’ pupal stage duration compared to “live bacteria” (x^2^ = 5.32, *P* = 0.021).

#### Egg to Adult Developmental Duration

The effect of *K. oxytoca* implementation on the whole immature developmental duration is shown in Supplementary Table S3 and **Figure S2C**. Focusing on males, bacterial addition, either “autoclaved” or “live,” statistically significant accelerated the immature developmental time compared to control treatment (log rank test x^2^ = 38.712, *P* < 0.001 for “autoclaved” and x^2^ = 39.277, *P* < 0.001 for “live”). The two bacterial treatments (“autoclaved” and “live”) exerted the same effect on males’ immature development (x^2^ = 0.011, *P* = 0.916). The bacteria fed females also completed their immature stages earlier than the females fed without bacteria. This effect was detected statistically only between the “autoclaved bacteria” and the control treatment (x^2^ = 4.852, *P* = 0.028) while the difference between “live bacteria” and control was not statistically significant, despite the observed trend (x^2^ = 3.518, *P* = 0.061). “Autoclaved” and “live bacteria” had the same impact on developmental duration of the immature stages (x^2^ = 0.131, *P* = 0.718).

### Effect of *K. oxytoca* as a Larval Diet Additive on Longevity under Food and Water Deprivation

**FIGURE 2:**
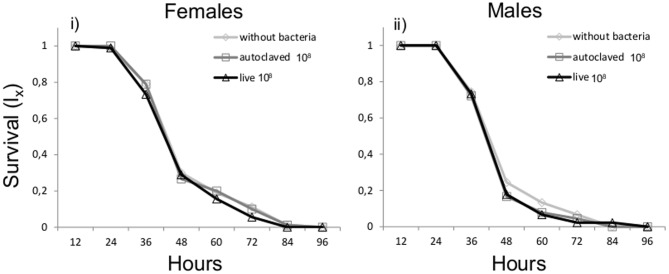
Survival under stress conditions with or without *K. oxytoca* incorporated in larval diet.

Cox regression analysis testing the effect of bacteria and sex on survival under stress conditions (water and food deprivation, light absence) revealed that neither bacteria treatment (Wald’ s test *t* = 1.31, df = 2, *P* = 0.519) nor sex (Wald’ s *t*-test = 2.59, df = 1, *P* = 0.108) were significant predictors of adult life span (**Figure 2**).

### Effect of *K. oxytoca* as a Larval Diet Additive on Flight Ability

**FIGURE 3:**
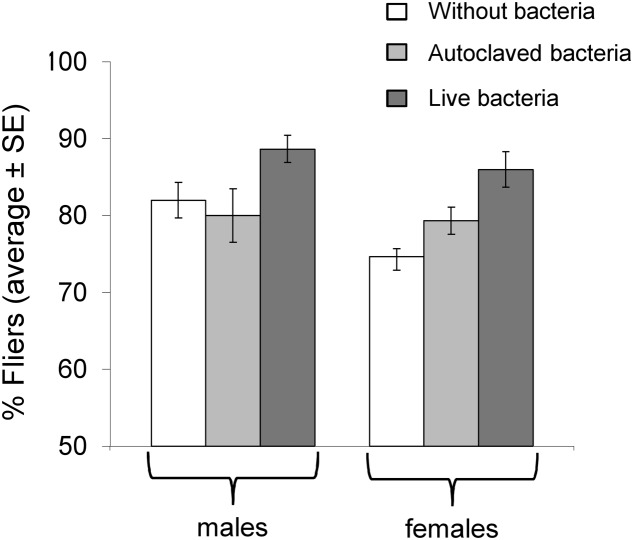
Flight ability in flies with and without *K. oxytoca* incorporated in larval diet.

Logistic regression analysis revealed that the bacteria addition was a significant predictor of the flight ability (Wald’s test *t* = 9.31, df = 2, *P* = 0.01). Specifically, insects fed in “live bacteria” diet presented better flight ability compared to both “autoclaved bacteria” fed (Wald’s test *t* = 6.29, df = 1, *P* = 0.012) and the control flies (Wald’s test *t* = 8.37, df = 1, *P* = 0.004). No difference was detected between the “autoclaved bacteria” and the control treatment (Wald’s test *t* = 0.16, df = 1, *P* = 0.689). Despite the tendency of females to display lower flight ability than males, statistical analysis did not detect significant differences between the sexes (Wald’s test *t* = 1.90, df = 1, *P* = 0.168) (**Figure 3**).

### Effect of *K. oxytoca* and *Enterobacter* sp. AA26 As Adult Diet Additives on Mating Competitiveness

**FIGURE 4:**
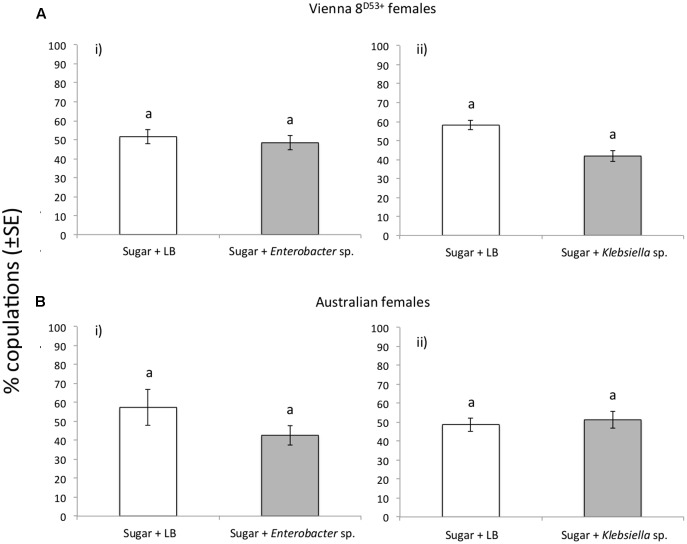
Mating success of the *Enterobacter* sp. AA26 and *K. oxytoca* fed, irradiated VIENNA 8^D53+^ males, when competed for **(A)** VIENNA 8^D53+^ GSS, and **(B)** Australian females. Columns marked on top with the same letter are not significantly different (*P* > 0.05).

Mating receptivity, as determined by the number of mates achieved in copulation tests irrespectively of the type of males (control, *Enterobacter* sp. and *Klebsiella* sp. fed), was statistically significant higher for VIENNA 8^D53+^ GSS females compared to Australian females (x^2^ = 44.70 and 40.92, respectively, df = 1, *P* < 0.001) (**Supplementary Figure S3**). Provisioning irradiated males with bacteria did not alter their mating competitiveness ability. The VIENNA 8^D53+^ males fed in Sugar/*Enterobacter* sp. AA26 diet displayed the same competitiveness ability with Sugar/LB fed males, when competed either for VIENNA 8^D53+^ or Australian females (x^2^ = 0.11, *P* = 0.734 for VIENNA 8^D53+^ females and x^2^ = 0.97, df = 1, *P* = 0.324 for Australian females). Likewise, *K. oxytoca* enhanced diet also did not change the mating competitiveness ability of the VIENNA 8^D53+^ males (x^2^ = 2.57 df = 1, *P* = 0.109 when competed for VIENNA 8^D53+^ females and x^2^ = 0.05, df = 1, *P* = 0.814 for Australian females) (**Figure 4**).

## Discussion

Our results demonstrate that the provision of *K. oxytoca* as a dietary supplement in the larval diet accelerated the immature development on VIENNA 8^D53+^. Specifically, feeding on “autoclaved” bacteria reduced both sexes immature developmental duration, whereas feeding on “live” bacteria accelerated males’ development. Although the same trend was evident for females as well, the statistical analysis did not detect it (marginally non-significant). We also recorded that “live” bacteria provision improves VIENNA 8^D53+^ flight ability. On the other hand, *K. oxytoca* enhanced larval diet did not exert any effect on pupae and adult recovery rates, sex ratio, and adult longevity under food and water deprivation. In respect to sterile male performance, the addition of *Enterobacter* sp. AA26 and *K. oxytoca* in their adult diet did not confer any effect on the mating competitiveness ability.

The incorporation of gut bacteria as probiotics in tephritid diets has recently received attention due to its great potential to improve the “quantitative” and “qualitative” parameters of the mass-reared strains. Most of the studies have been performed in medfly and specifically in the VIENNA 8 GSS (Niyazi et al., 2004; Ben Ami et al., 2010; Gavriel et al., 2011; Hamden et al., 2013; Augustinos et al., 2015) and very few in other species, such as *B. oleae* (Sacchetti et al., 2014), *B. tryoni* (Meats et al., 2009), *Z. tau* (Khan et al., 2014), and *A. obliqua* (Rull et al., 2015).

In the present study, the well-characterized VIENNA 8^D53+^ medfly laboratory strain was used. This strain has been evaluated in the preceding study of our group, whereas other relative probiotic studies have also been conducted with medfly strains generated from the same parental medfly lines (Niyazi et al., 2004; Ben Ami et al., 2010; Gavriel et al., 2011; Hamden et al., 2013). The *K. oxytoca* isolate used in our experiments is a naturally occurring medfly gut symbiont. The same isolate was used also by Ben Ami et al. (2010) and Gavriel et al. (2011), whereas Hamden et al. (2013) evaluated *Klebsiella* sp. isolate of unknown origin (combined with other isolates of unknown origin). The *Enterobacter* sp. AA26 isolate used as an adult diet supplement in the current study is also a naturally occurring medfly gut symbiont and the same with the one used before as a larval diet supplement by Augustinos et al. (2015).

### Probiotic Supplements in Larval Diet

To our knowledge, there are only two studies that have used probiotic supplements in larval diets. First, Hamden et al. (2013) tested a probiotic cocktail of three different Enterobacteriaceae (*Enterobacter* sp., *K. pneumoniae*, and *C. freundii*), not naturally present in medfly. This study reported various beneficial effects in pupal weight, flight ability of irradiated males, adult size, mating competitiveness of irradiated males and sperm transfer. On the other hand, Augustinos et al. (2015), using the *Enterobacter* sp. AA26 (naturally present in medfly’s gut) did not detect any significant effect of the probiotic diet on pupal weight, sex ratio, adult longevity under stress (food and water deprivation) and mating competitiveness but found a significant effect on recovery rates (increase) and immature developmental stages duration (reduction). They also reported that bacteria feeding tended to improve adults flight ability, although the differences between probiotic and control treatments was not statistically significant. In the current study, the addition of *K. oxytoca* in larval diet did not affect either recovery rates (as *Enterobacter* sp. AA26 did before) or longevity under stress conditions but significantly reduced immature developmental stages duration and had a clearer positive effect, compared to *Enterobacter* sp. AA26, on flight ability. To summarize, data from different studies can be regarded as agreeing for specific effects (reduction of immature developmental time) or rather controversial for other parameters (such as recovery rates and mating competitiveness).

### Probiotic Supplements in Adult Diet

More studies have utilized bacteria as supplement in adult diets of tephritids. Like larval diet trials, most of them have also been performed in medfly and, more specifically, on the VIENNA 8 GSS. First, Niyazi et al. (2004) inoculated adult diet with a mix of *Enterobacter agglomerans* and *K. pneumoniae* and found beneficial effects on the males sexual signaling, mating competitiveness and lifespan of the irradiated males. Later on, Behar et al. (2008) used an Enterobacteriaceae mix as adult diet supplement and found a positive effect on adults’ longevity. A follow-up study showed that the supplement of *K. oxytoca* affects mating latency time (Ben Ami et al., 2010) and, later, the addition of the same *K. oxytoca* in adult diet was shown to increase the mating competitiveness of irradiated males, reduce the female mating receptivity and affect female longevity under stress conditions (Gavriel et al., 2010). In our study, the addition of *Enterobacter* sp. AA26 or *K. oxytoca* in the adult diet did not have any clear effect on irradiated males mating success, although the same bacterial isolate was also used in the study of Gavriel et al. (2011). In the present study, we tried to detect any beneficial effect of the *Enterobacter* sp. AA26 and *K. oxytoca* on sterile male mating competitiveness, when provided as adult diet supplement (live bacteria). We did not manage to detect the beneficial effects reported by Gavriel et al. (2011) who tested the mating competitiveness ability of nutritionally restricted males (only sugar fed). To exclude the possibility that any positive effect derives from the addition of nutrients from the bacteria growing medium, an equal volume of LB medium was added in the control diet. Combining the results of these studies, it is possible that the beneficial effect of probiotic diets may be maximized when diet is poor. The fact that in Gavriel et al. (2011), the beneficial effect on mating competitiveness was documented only when live bacteria were used (in contrast with autoclaved) shows that data interpretation can be challenging and goes beyond nutritional value.

Besides medfly, very few studies are available for other tephritids. In *B. tryoni*, the provision of *Klebsiella* sp. did not have any beneficial effect either on egg production or in sexual attraction, in an experiment performed in more than one generation during laboratory domestication (Meats et al., 2009). In *Z. tau*, the provision of *Proteus* sp. and *Klebsiella* sp. as adult diet supplements did not show any effect on the ovariole number and females fecundity (Khan et al., 2014). More recently, the provision of a naturally occurring gut symbiont of *B. oleae* (*Pseudomonas putida*) did not confer any advantage regarding adult longevity but was beneficial for female fecundity when tested under dietary restricted conditions (Sacchetti et al., 2014). Finally, in *A. obliqua*, adult feeding with an uncharacterized bacterial cocktail deriving from total gut extract did not have any positive effect on adult longevity (Rull et al., 2015).

Some of the positive effects of *K. oxytoca-*enriched diets, such as the reduction of immature developmental time, were evident no matter if it was provided as ‘autoclaved’ or ‘live’ bacteria. On the other hand, the positive effect on flight ability was demonstrated only when *K. oxytoca* was provided as a ‘live’ supplement (although its viability on the food was not tested). The different effects or the different ‘extent’ of the positive effects of ‘autoclaved’ and ‘live’ supplements show that these two approaches have different beneficial values. Autoclaved bacteria can probably provide nutrients and mainly act as an additional food source, although non-nutritional beneficial effects of dead probiotics (or ‘paraprobiotics’) cannot be excluded (Adams, 2010; de Almada et al., 2016). On the other hand, live bacteria could establish, modify the existing git microbial community and have a more discrete role both for nutrition and development. Follow up experiments regarding the viability of bacteria in larval diet and their localization/quantification in the insect’s gut during development can provide more insight on how probiotic diets actually work.

### Origin of Controversy

Comparing findings from different studies, since experiments are conducted with different insect species or strains, using different bacteria species/strains and fed on different stages, is not easy. Not all studies answer to the same questions, since they try to shed light to different parameters that are considered important in SIT (such as rearing productivity or biological quality – related factors). The methodological set up can be quite different as well. Additionally, data interpretation is influenced by what each lab defines as standard (or control) rearing conditions. As an example, in the larval diet, different substrates are being used that can influence the nutritional value of the “control” diet, and the crowding conditions in rearing may also vary. These aspects could strongly affect the results and consequently the probiotic role assigned to the bacteria. For example, certain nutrients offered by bacteria may be important in some artificial diets but not in others. Interestingly, the same (or similar) bacterial taxa do not consistently have the same effects. Over the last decade, many studies have tried to shed light to the characterization of gut symbiotic communities of tephritids and found that gut symbiotic communities, although relatively poor, show great variations in the different developmental stages at genus level (Ben Ami et al., 2010; Aharon et al., 2012; Andongma et al., 2015; Augustinos et al., 2015). This may reflect the development of preferential relationships and may explain why specific bacteria do not ‘retain’ their beneficial effects when provided in different developmental stages.

## Conclusion

The different probiotic studies performed up to now are quite promising regarding the enhancement of SIT application. Improvements could derive from shortening of the developmental time of immature stages (therefore less space would be required), further enhancement of protandry (that could be the basis for more accurate sex separation systems), increased adult recovery numbers (that would reduce the cost of SIT) and males that perform better in the field (better flight ability and mating competitiveness, that would also reduce the cost and enhance the effectiveness of SIT). However, although encouraging, they are far from conclusive and even more far away from becoming default practices in mass rearing facilities. Even if live bacteria may seem to be more efficient than autoclaved ones according to some studies, the addition of bacteria in autoclaved form is rather more realistic due to the minor amendments required in the currently used rearing protocols. Future studies should further confirm the actual impact of probiotic provision on mass-rearing by developing an effective and generally accepted implementation protocol, including the cost benefit analyses of industrial production of probiotics for mass production of sterile insects.

## Author Contributions

GK designed and performed experiments, analyzed data and drafted part of the manuscript. AA designed and performed experiments, analyzed data and drafted part of the manuscript. CC conceived and designed experiments, interpreted the data and critically revised the manuscript. KB conceived and designed experiments, interpreted the data and critically revised the manuscript. All authors approved the final version of the manuscript and agreed to be accountable for all aspects of the work in ensuring that questions related to the accuracy or integrity of any part of the work are appropriately investigated and resolved.

## Conflict of Interest Statement

The authors declare that the research was conducted in the absence of any commercial or financial relationships that could be construed as a potential conflict of interest.

## Footnotes

1 https://nucleus.iaea.org/sites/naipc/dirsit/SitePages/Forms/AllPages.aspx

## Supplementary Material

**FIGURE S1 |** Sex ratio determination. (i) considering each one of the three *Klebsiella oxytoca* concentrations as different treatments, or (ii) irrespective of the *K. oxytoca* concentration. Columns headed with the same letter are not significantly different (*P* > 0.05).

**FIGURE S2 |** Daily allocation of the immature stages duration for the three *K. oxytoca* treatments. **(A)** Percentage of total number of pupae recovered per day (number of days after egg laying), **(B)** percentage of total number of adults recovered per day (number of days after pupation), **(C)** total immature stages duration (number of days after egg laying).

**FIGURE S3 |** Mating receptivity of the Australian and the Vienna 8^D53+^ females, as determined by the number of females that achieved to mate (% mated females). Columns marked on top with the same letter are not significantly different (*P* > 0.05).
