# Supplementary figures and images for: Medfly Gut Microbiota and Enhancement of the Sterile Insect Technique: Similarities and Differences of Klebsiella oxytoca and Enterobacter sp. AA26 Probiotics during the Larval and Adult Stages of the VIENNA 8D53+ Genetic Sexing Strain
Source: Front Microbiol. 2017 Oct 27;8:2064. doi: 10.3389/fmicb.2017.02064 (PMC5663728; doi:10.3389/fmicb.2017.02064)

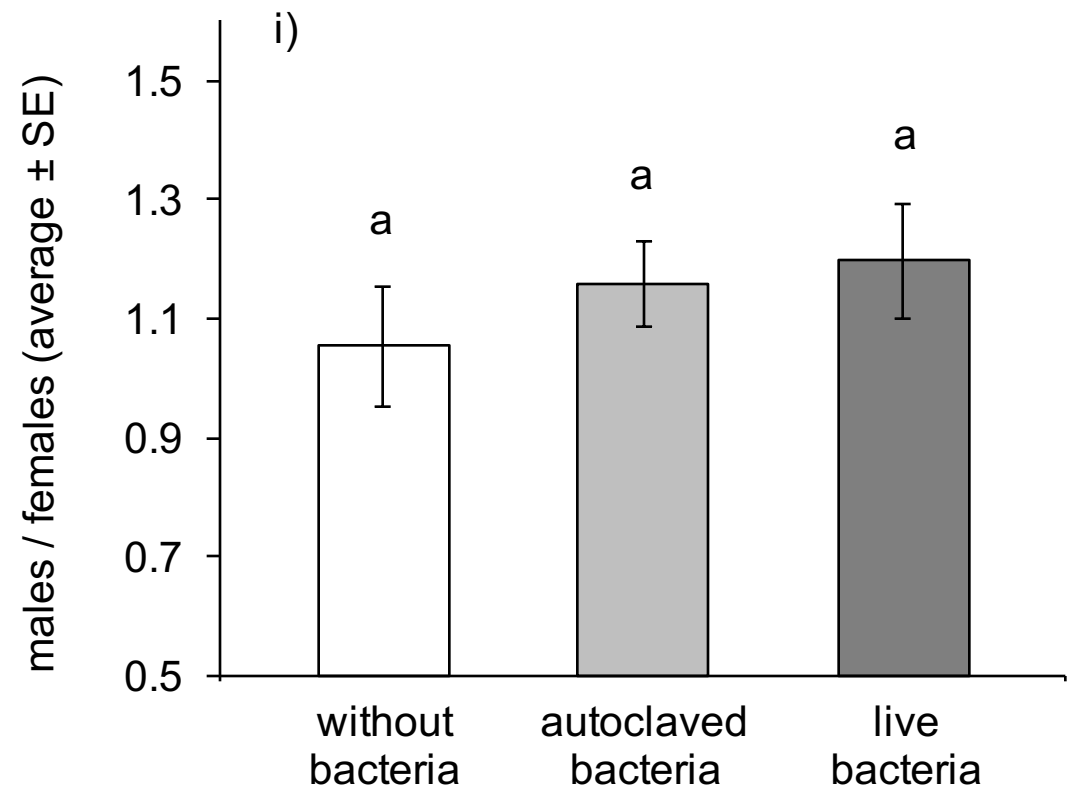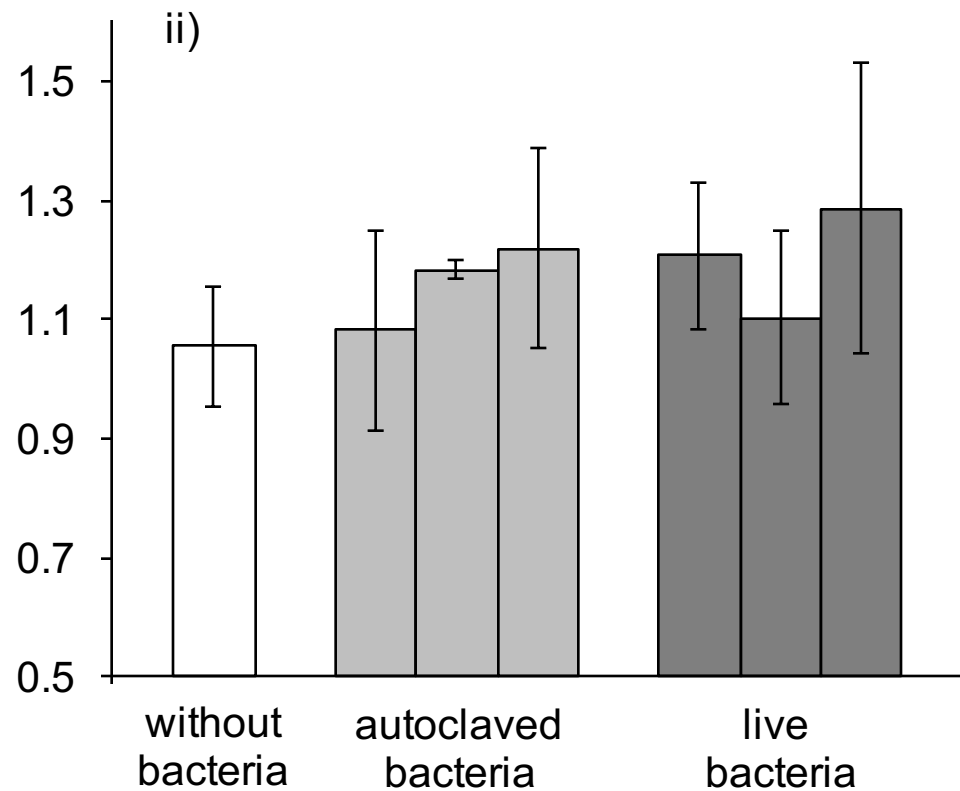

Supplement: Supplementary file 1 [file Image_1.pdf]

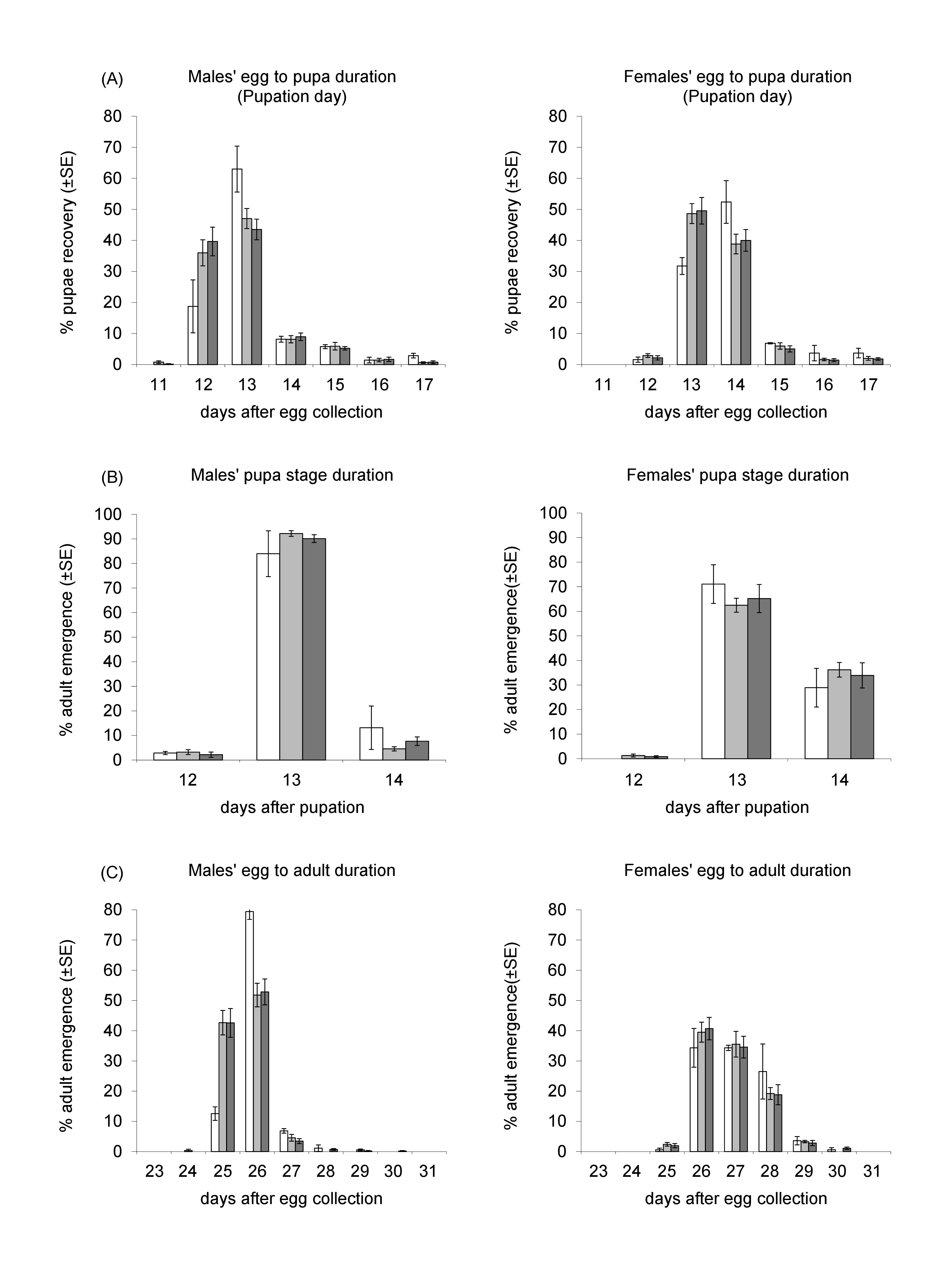

Supplement: Supplementary file 2 [file Image_2.TIFF]

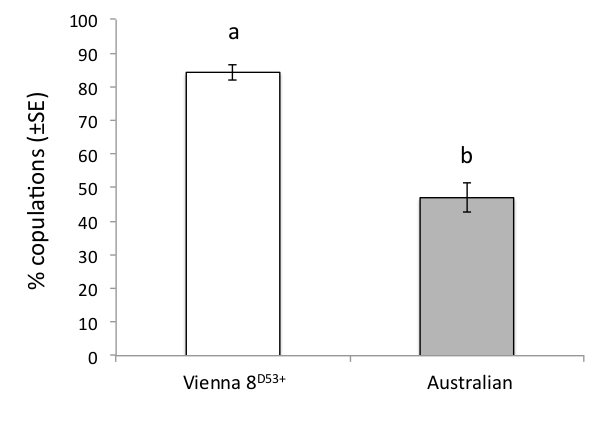

Supplement: Supplementary file 3 [file Image_3.TIF]
