# Supplementary material for: Medfly Gut Microbiota and Enhancement of the Sterile Insect Technique: Similarities and Differences of Klebsiella oxytoca and Enterobacter sp. AA26 Probiotics during the Larval and Adult Stages of the VIENNA 8D53+ Genetic Sexing Strain
Source: Front Microbiol. 2017 Oct 27;8:2064. doi: 10.3389/fmicb.2017.02064 (PMC5663728; doi:10.3389/fmicb.2017.02064)
Supplement: Supplementary file 4 [file Table_1.DOCX]

**Table S1.** *K.oxytoca* enriched larval diet and egg to pupae developmental duration.

| Treatment | n | Mean (days) ± SE | Kaplan-Meier / log-rank (Mantel-Cox) |
| --- | --- | --- | --- |
| Males |  |  |  |
| W | 208 | 13.168±0.072 | W vs A: x^2^ = 13.98, P < 0.001 |
| A | 625 | 12.893±0.039 | W vs L: x^2^ = 15.68, P < 0.001 |
| L | 648 | 12.877±0.039 | A vs L: x^2^ = 0.17, P = 0.680 |
| Females |  |  |  |
| W | 189 | 13.905±0.070 | W vs A: x^2^ = 15.38, P < 0.001 |
| A | 551 | 13.608±0.037 | W vs L: x^2^ = 17.75, P < 0.001 |
| L | 555 | 13.595±0.035 | A vs L: x^2^ = 0.07, P = 0.795 |

*W = without bacteria, A = autoclaved bacteria, L = live bacteria diets*
