# Supplementary material for: Medfly Gut Microbiota and Enhancement of the Sterile Insect Technique: Similarities and Differences of Klebsiella oxytoca and Enterobacter sp. AA26 Probiotics during the Larval and Adult Stages of the VIENNA 8D53+ Genetic Sexing Strain
Source: Front Microbiol. 2017 Oct 27;8:2064. doi: 10.3389/fmicb.2017.02064 (PMC5663728; doi:10.3389/fmicb.2017.02064)
Supplement: Supplementary file 5 [file Table_2.DOCX]

**Table S2.** *K. oxytoca* enriched larval diet and pupal stage duration.

| Treatment | n | Mean (days) ± SE | Kaplan-Meier / log-rank (Mantel-Cox) |
| --- | --- | --- | --- |
| Males |  |  |  |
| W | 175 | 13.103±0.029 | W vs A: x^2^ = 11.22, P = 0.001 |
| A | 525 | 13.013±0.012 | W vs L: x^2^ = 2.99, P = 0.083 |
| L | 547 | 13.055±0.013 | A vs L: x^2^ = 5.32, P = 0.021 |
| Females |  |  |  |
| W | 166 | 13.289±0.035 | W vs A: x^2^ = 2.13, P = 0.145 |
| A | 453 | 13.349±0.024 | W vs L: x^2^ = 1.05, P = 0.306 |
| L | 457 | 13.330±0.023 | A vs L: x^2^ = 0.38, P = 0.537 |

*W = without bacteria, A = autoclaved bacteria, L = live bacteria diets*
