# Supplementary material for: Medfly Gut Microbiota and Enhancement of the Sterile Insect Technique: Similarities and Differences of Klebsiella oxytoca and Enterobacter sp. AA26 Probiotics during the Larval and Adult Stages of the VIENNA 8D53+ Genetic Sexing Strain
Source: Front Microbiol. 2017 Oct 27;8:2064. doi: 10.3389/fmicb.2017.02064 (PMC5663728; doi:10.3389/fmicb.2017.02064)
Supplement: Supplementary file 6 [file Table_3.DOCX]

**Table S3.** *K. oxytoca* enriched larval diet and immature developmental stages duration

| Treatment | n | Mean (days) ± SE | Kaplan-Meier / log-rank (Mantel-Cox) |
| --- | --- | --- | --- |
| Males |  |  |  |
| W | 175 | 25.966±0.037 | W vs A: x^2^ = 38.71, P < 0.001 |
| A | 525 | 25.629±0.028 | W vs L: x^2^ = 39.28, P < 0.001 |
| L | 547 | 25.636±0.027 | A vs L: x^2^ = 0.01, P = 0.916 |
| Females |  |  |  |
| W | 166 | 27.000±0.071 | W vs A: x^2^ = 4.85, P = 0.028 |
| A | 453 | 26.815±0.042 | W vs L: x^2^ = 3.52, P = 0.061 |
| L | 457 | 26.832±0.043 | A vs L: x^2^ = 0.131, P = 0.718 |

*W = without bacteria, A = autoclaved bacteria, L = live bacteria diets*
